# Professor W. H. Eames

**Published:** 1894-05

**Authors:** 


### PROFESSOR W. H. EAMES.

On another page will be found the announcement of the death of this prominent worker in the profession, accompanied by a sketch of his life.

The blow has fallen with a shock to his friends in the East, and it is difficult to realize that we will never again meet with him in the work in which he was ever an earnest and most efficient laborer.

It has been our pleasure to meet with him yearly in our several annual conventions, and especially in connection with the National Association of Dental Faculties. In this body he exercised a powerful influence. His clear logical mind made him a valuable colaborer in the work, always ready to unravel the intricacies of difficult questions and place them in a light to be readily understood. He was honored in this organization by being made its presiding officer.

While a resident in the West, and energetic there in associations, editorial work, and college labor, his activity passed beyond sectional limits and grasped the needs of the entire profession. He was broad in his intellectual force, genial in companionship, and thoroughly in harmony with the progressive demands of the hour.

Death has gathered a rich harvest in the past few years, but while the silent reaper has taken many from our ranks whose places have never been filled, few of these will be more sincerely mourned by his co-workers than this faithful exponent of the best life of his profession.

